# Electromechanical Modeling of a Piezoelectric Vibration Energy Harvesting Microdevice Based on Multilayer Resonator for Air Conditioning Vents at Office Buildings

**DOI:** 10.3390/mi10030211

**Published:** 2019-03-26

**Authors:** Ernesto A. Elvira-Hernández, Luis A. Uscanga-González, Arxel de León, Francisco López-Huerta, Agustín L. Herrera-May

**Affiliations:** 1 Micro and Nanotechnology Research Center, Universidad Veracruzana, Calzada Ruiz Cortines 455, Boca del Río, Veracruz 94294, Mexico; aelvirah@hotmail.com; 2 Faculty of Mechanical and Electrical Engineering, Universidad Veracruzana, Xalapa, Veracruz 91000, Mexico; luscanga89@gmail.com; 3 CONACYT-Centro de Investigación en Química Aplicada, Boulevard Enrique Reyna 140, Saltillo Coahuila 25294, Mexico; arxel.deleon@ciqa.edu.mx; 4 Facultad de Ingeniería Eléctrica y Electrónica, Universidad Veracruzana, Calzada Ruiz Cortines 455, Boca del Río, Veracruz 94294, Mexico; frlopez@uv.mx; 5 Maestría en Ingeniería Aplicada, Facultad de Ingeniería de la Construcción y el Hábitat, Universidad Veracruzana, Calzada Ruíz Cortines 455, Boca del Río, Veracruz 94294, Mexico

**Keywords:** electromechanical modeling, Euler-Bernoulli beam theory, finite element method, microdevice, multilayer beams, piezoelectric energy harvesting, Rayleigh method, resonator, Macaulay method

## Abstract

Piezoelectric vibration energy harvesting (pVEH) microdevices can convert the mechanical vibrations to electrical voltages. In the future, these microdevices can provide an alternative to replace the electrochemical batteries, which cause contamination due to their toxic materials. We present the electromechanical modeling of a pVEH microdevice with a novel resonant structure for air conditioning vents at office buildings. This electromechanical modeling includes different multilayers and cross-sections of the microdevice resonator as well as the air damping. This microdevice uses a flexible substrate and it does not include toxics materials. The microdevice has a resonant structure formed by multilayer beams and U-shape proof mass of UV-resin (730 μm thickness). The multilayer beams contain flexible substrates (160 μm thickness) of polyethylene terephthalate (PET), two aluminum electrodes (100 nm thickness), and a ZnO layer (2 μm thickness). An analytical model is developed to predict the first bending resonant frequency and deflections of the microdevice. This model considers the Rayleigh and Macaulay methods, and the Euler-Bernoulli beam theory. In addition, the electromechanical behavior of the microdevice is determined through the finite element method (FEM) models. In these FEM models, the output power of the microdevice is obtained using different sinusoidal accelerations. The microdevice has a resonant frequency of 60.3 Hz, a maximum deflection of 2.485 mm considering an acceleration of 1.5 m/s^2^, an output voltage of 2.854 V and generated power of 37.45 μW with a load resistance of 217.5 kΩ. An array of pVEH microdevices connected in series could be used to convert the displacements of air conditioning vents at office buildings into voltages for electronic devices and sensors.

## 1. Introduction

Most of the electrochemical batteries include toxic materials that can cause environment contamination [1,2]. These batteries have large size and limited operating time, which complicate their used for microdevices of Internet of Things (IoT). In the future, the number of self-powered microdevices will significantly increase due to IoT. For this, the conventional batteries will must be replaced by novel power sources. An alternative solution is the development of energy harvesting devices that can convert the environment energy (e.g., mechanical vibrations, thermal energy, solar radiation, wind energy and movement of the human body) into electrical energy [3,4,5,6,7,8,9]. For instance, the kinetic energy caused by the mechanical vibrations in the environment could be transformed into electrical energy through vibration energy harvesting (VEH) devices. These devices can be classified according to their transduction principle: electromagnetic, electrostatic, and piezoelectric [5]. Electromagnetic VEH devices do not need an external voltage source and can operate at low frequencies. However, the electromagnetic VEH devices require a post-processing technique to deposit their magnetic material. On the other hand, electrostatic VEH devices generate suitable output voltage (e.g., up 10 V) and have good integration with microelectromechanical systems (MEMS) technology. While these devices need complex electronic circuits and correct alignment between their plates. Finally, piezoelectric vibration energy harvesting (pVEH) devices can be developed using microfabrication process and can provide output voltages without requiring external voltage sources.

Commonly, mechanical vibrations in the environment have frequencies below 100 Hz. Thus, the pVEH devices should operate close to this frequency to increase the voltage obtained of the mechanical vibrations [10]. Prušáková et al. [11] fabricated a pVEH microdevice based on a multilayer cantilever with a silicon substrate, two aluminum electrodes and a ZnO layer. This microdevice can generate 0.975 V at resonant frequency of 592 Hz; however, this frequency value is higher than the frequency of the mechanical vibrations in the environment. On the other hand, Chang et al. [12] designed a pVEH cantilever composed by a ZnO film deposited on a flexible stainless-steel substrate, a Cu layer and an additional mass (0.57 g). This cantilever at resonance (75 Hz) can convert the wind energy into electrical energy, generating a voltage of 10.5 V. In other research, Wang and Du [13] fabricated two pVEH microdevices using a silicon substrate, a SiO_2_ layer, two Au/Ti electrodes and a ZnO layer. These microdevices have similar natural frequency (1300.1 Hz) and can generate 2.06 V and 1.77 V, respectively, for mechanical vibrations with an acceleration of 10 m/s^2^. In order to generate the maximum power, the pVEH microdevices must be designed to oscillate at resonance with the same frequency of the mechanical vibration source in the environment. Thus, for each application must be developed a pVEH microdevice that optimizes its output power without overcome the rupture stresses of their materials. Many office buildings use air conditioning systems that generate mechanical vibrations in their vents. To take advantage of the mechanical vibrations in air conditioning vents, we designed a pVEH microdevice that can be installed on these vents to generate an output voltage of 2.854 V and output power of 37.45 mW with a load resistance of 217.5 kΩ. This design contains a novel resonant structure based on flexible substrate and it does not include toxics materials. The resonant structure is composed by multilayer beams and U-shape proof mass of UV-resin (730 μm thickness). The resonant structure has a flexible substrate (160 μm thickness) of polyethylene terephthalate (PET), two aluminum electrodes (100 nm thickness), and a zinc oxide (ZnO) layer (2 μm thickness). The electromechanical modeling of the microdevice is obtained using the Rayleigh and Macaulay methods, Euler-Bernoulli beam theory and finite element method (FEM) models. This modeling takes in account the different multilayers and cross-sections of the microdevice resonator as well as the air damping. Additionally, the electromechanical behavior of the microdevice is studied considering five different accelerations amplitudes. The proposed microdevice can be used to convert the mechanical vibrations of air conditioning vents at office buildings into electrical energy.

This paper is organized as follows: Section 2 includes the modeling of the pVEH microdevice to determine its first bending resonant frequency and deflections. Section 3 describes the results and discussion about the mechanical and electrical behavior of the pVEH microdevice. Finally, the conclusions and future work are indicated.

## 2. Modeling of the pVEH Microdevice

In this section, we present the modeling of a pVEH microdevice to determine its mechanical and electrical. The microdevice modeling is obtained using the Rayleigh and Macaulay methods, Euler-Bernoulli beam theory, and finite element method (FEM) models. 

### 2.1. Design

**Figure 1:**
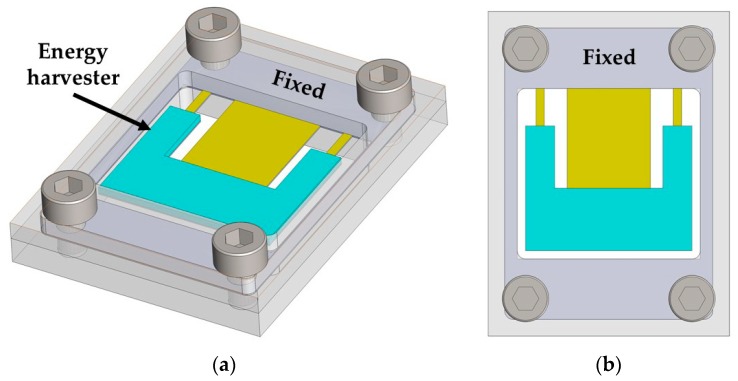
3D design of the piezoelectric vibration energy harvesting (pVEH) microdevice for air conditioning vents at office buildings, considering isometric (**a**) and plant (**b**) views.

**Figure 2:**
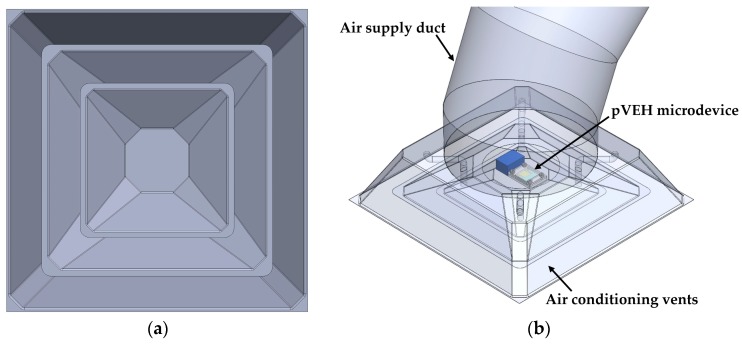
(**a**) Front and (**b**) isometric views of the air conditioning vents at office building with a pVEH microdevice.

**Figure 3:**
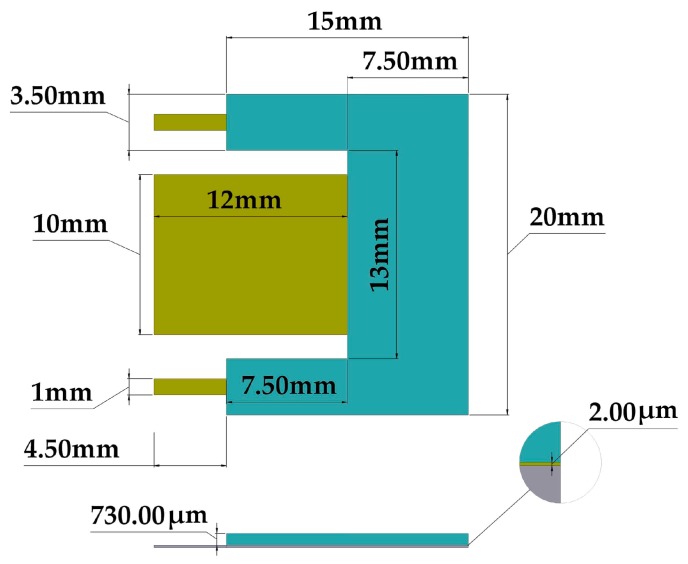
Dimensions of the main components of the pVEH microdevice.

Figure 1 shows the 3D design of the pVEH microdevice, which is connected to a fixed support. This pVEH microdevice can be mounted on the air conditioning vents at office buildings (Figure 2), which achieve mechanical vibrations close to 60 Hz with acceleration of 1.5 m/s^2^ [14]. The microdevice contains a resonator of multilayer beams (the main dimensions are depicted in Figure 3) with a PET substrate (160 μm thickness), two aluminum electrodes (100 nm thickness), a ZnO layer (2 μm thickness), and U-shape proof mass of UV-resin (730 μm thickness). The U-shape of the seismic mass of the microdevice is selected to decrease its stiffness without increasing the length. In addition, ZnO layer does not contain contaminate materials as the PZT (lead zirconate titanate) [15,16]. The ZnO layer has a high tensile strength and it does not need a polarization process after it is deposited [16].

The aluminum electrodes are located at the bottom and top surface of the piezoelectric (ZnO) layer. The aluminum material has good adhesion and coupling of its lattice constants with those of ZnO [17]. This resonator can oscillate in its first bending vibration mode to achieve maximum out-plane displacements, which will increase its output voltage.

The main damping source of the microdevice resonator is due to the air damping, which can be determined through its the quality factor (*Q*). This factor is affected by the air pressure around the resonator [18]. This factor is the ratio between the total energy stored in the resonator (*E_T_*) and the energy factor lost per cycle (*E_C_*) caused by the damping source:

(1) $$Q=2\pi \frac{E_{T}}{E_{C}}$$

The damping ratio (ζ) of the resonator is related with its quality factor and can be calculated by

(2) $$\zeta =\frac{1}{2Q}$$

To calculate the quality factor of the resonator due to the air damping, we consider the resonant structure as a simple equivalent cantilever (width *b*, thickness *h*, and length *L_e_*) formed by a PET substrate with a proof mass attached to its free end. For this case, the quality factor associated with the air damping at atmospheric pressure (*Q_a_*) can be obtained using the Blom model [19]:

(3) $$Q_{a}=\frac{f_{r}\rho_{p}bhL_{e}}{3\mu R\left(1+R/\beta \right)}$$

with

(4) $$\beta =\sqrt{\frac{\mu }{\pi \rho_{a}f_{r}}}$$

(5) $$R=\sqrt{\frac{bL_{e}}{\pi }}$$

where *f_r_* is the resonant frequency of the microdevice, *ρ_p_* is the PET density, *μ* and *ρ_a_* the viscosity and density of the air, respectively.

### 2.2. Analytical Modeling

To determine the first bending resonant frequency of the microdevice structure, we employed the Rayleigh and Macaulay methods, as well as the Euler-Bernoulli beam theory. Based on the Rayleigh method, the resonant frequency of a cantilever can be obtained through the maximum potential energy (*P_m_*) and kinetic energy (*K_m_*) [20,21]:

(6) $$P_{m}=\frac{1}{2}{\int_{0}^{L}EI(x)\left(\frac{\partial^{2}y(x)}{\partial x^{2}}\right)}^{2}dx$$

(7) $$K_{m}=\frac{{\left(2\pi f\right)}^{2}}{2}\int_{0}^{L}\rho A(x)y^{2}(x)dx$$

where *y*(*x*) is the bending displacement at a given point along *x*-axis of the cantilever, *f* is the resonator frequency, *L, A, E, I,* and *ρ* are the length, cross-section area, Young’s modulus, moment of inertia, and density of the cantilever, respectively. 

Thus, the resonant frequency (*f_rc_*) of a cantilever can be calculated considering the energy conservation equation (*P_m_* = *K_m_*). By substituting Equations (6) and (7) into energy conservation equation, the resonant frequency is given by:

(8) $$f_{rc}=\frac{1}{2\pi }\sqrt{\frac{\int_{0}^{L}EI(x){\left(\frac{\partial^{2}y(x)}{\partial x^{2}}\right)}^{2}dx}{\int_{0}^{L}\rho A(x)y^{2}(x)dx}}$$

**Figure 4:**
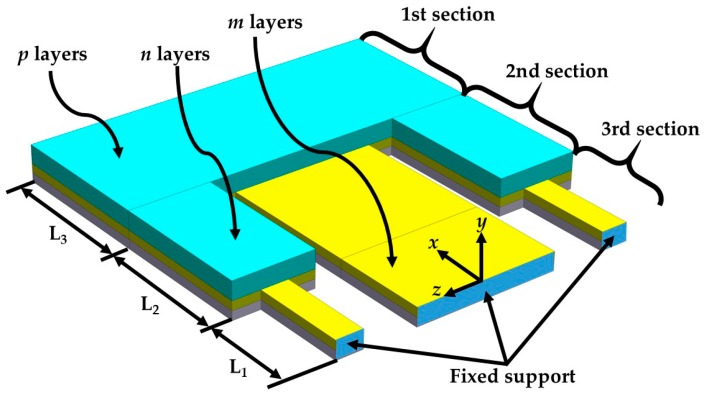
Schematic of the pVEH microdevice equivalent resonator, which is divided in three different cross-sections.

**Figure 5:**
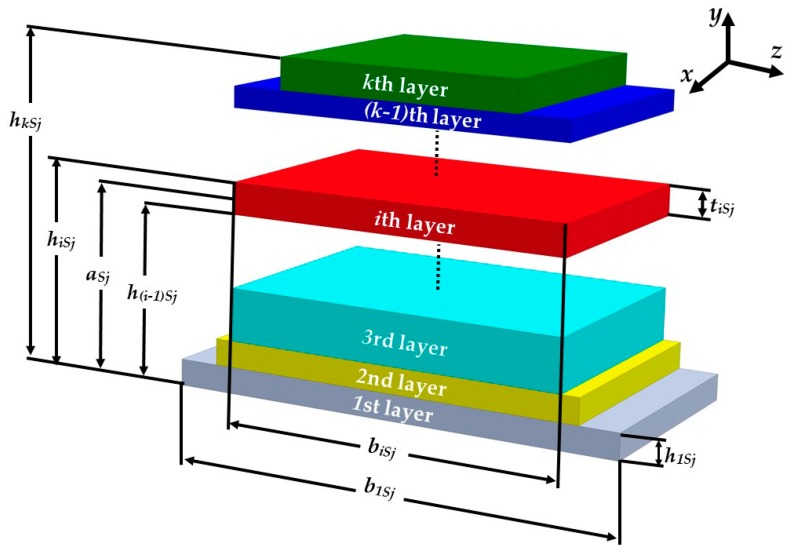
View of the nomenclature used to determine the elastic centroid (*a_sj_*) of the *j*th cross-section of pVEH microdevice equivalent resonator.

**Figure 6:**
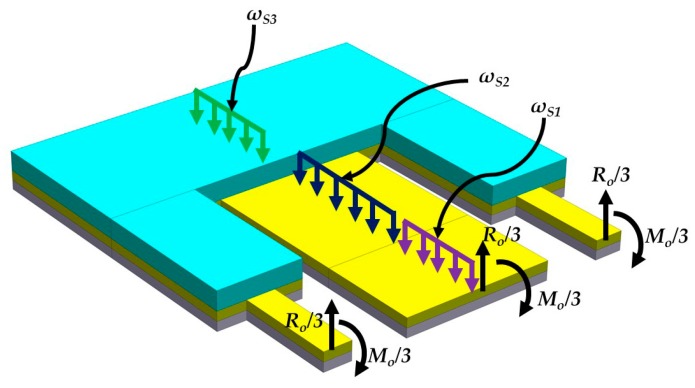
View of the uniformly distributed loads (*ω_Sj_*), bending moments (*M_o_*) and reaction loads (*R_o_*) of the pVEH microdevice equivalent resonator.

The microdevice resonator has different multilayers and cross-sections; therefore, the equivalent bending stiffness and elastic centroid of the microdevice resonator are determined. For this, we consider a microdevice equivalent resonator with three different sections that include a PET substrate, a ZnO layer and a seismic mass of UV-resin (see Figure 4). In the equivalent resonator was neglected both aluminum electrodes due to their small thickness (100 nm). The sections of the equivalent resonator contain *m*th, *n*th and *p*th layers, which are symmetric with respect to the *xy*-plane. In addition, the *j*th nomenclature describes each one of the three sections of the microdevice. Figure 5 illustrates the nomenclature to calculate the elastic centroid of the *j*th cross-section of the equivalent resonator. For this nomenclature, parameter *h_i_*_S*j*_ represents the distance between the bottom and top plane of the first layer of the *j*th cross-section. Additionally, *t_i_*_S*j*_ and *b_i_*_S*j*_ indicate the thickness and width of the *i*th layer located in the *j*th section. Figure 6 depicts the uniformly distributed loads (*ω_S_*_j_) in the *j*th cross-section, bending moments (*M_o_*), and reaction loads (*R_o_*) at the fixed support of the equivalent resonator.

For the analytical modeling of the equivalent resonator, we assume that the plane sections of layers do not deform. In addition, the residual stress and transverse shear strain are neglected. Further, the layers are considered isotropic and homogeneous.

The elastic centroid (*a_Sj_*) of each section of the equivalent resonator can be determined as [22]:

(9) $$a_{Sj}=\frac{{\left(ES\right)}_{Sj}}{{\left(EA\right)}_{Sj}}=\frac{\iint_{A_{Sj}}E_{Sj}y_{Sj}(x)dydz}{\iint_{A_{Sj}}E_{Sj}dydz}=\frac{1}{2}\frac{\sum_{i=1}^{q}E_{iSj}b_{iSj}t_{iSj}\left(h_{iSj}+h_{(i-1)Sj}\right)}{\sum_{i=1}^{q}E_{iSj}b_{iSj}t_{iSj}}$$

where *E_iSj_* is the Young’s modulus of the *i*th layer located in the *j*th section, *A_Sj_* is the domain in the *j*th section, *t_i_*_S*j*_ is the thickness of the *i*th layer placed in the *j*th section, *h*_(*i*−1)S*j*_ is the distance between the bottom surface of the first layer and the top surface of the (*i* − 1)th layer situated at the *j*th section, and *b_i_*_S*j*_ is the width of the *i*th layer collocated in the *j*th section.

The bending stiffness of equivalent resonator can be calculated by:

(10) $${\left(EI_{z}\right)}_{Sj}={\sum_{i}^{q}\left(E_{i}I_{zi}\right)}_{S_{j}}=\iint_{A_{Sj}}E_{Sj}y_{Sj}(x)dy=\frac{1}{3}\sum_{i=1}^{q}E_{iSj}b_{iSj}\left[{\left(h_{iSj}-a_{Sj}\right)}^{3}-{\left(h_{(i-1)Sj}-a_{Sj}\right)}^{3}\right]$$

To obtain the maximum kinetic energy (*K_m_*) and potential energy (*P_m_*) of the equivalent resonator, we use the following expressions:

(11) $$P_{m}=\frac{1}{2}{\left(EI_{z}\right)}_{S_{1}}\int_{0}^{L_{1}}{\left(\frac{\partial^{2}y_{S_{1}}(x)}{\partial x^{2}}\right)}^{2}dx+\frac{1}{2}{\left(EI_{z}\right)}_{S_{2}}\int_{L_{1}}^{L_{12}}{\left(\frac{\partial^{2}y_{S_{2}}(x)}{\partial x^{2}}\right)}^{2}dx+\frac{1}{2}{\left(EI_{z}\right)}_{S_{3}}\int_{L_{12}}^{L_{123}}{\left(\frac{\partial^{2}y_{S_{3}}(x)}{\partial x^{2}}\right)}^{2}dx$$

(12) $$\begin{array}{ll} \frac{K_{m}}{\theta^{2}}= & \frac{1}{2}\left(\sum_{i=1}^{m}\rho_{iS_{1}}b_{iS_{1}}t_{iS_{1}}\right)\int_{0}^{L_{1}}{\left(y_{S_{1}}(x)\right)}^{2}dx+\frac{1}{2}\left(\sum_{i=1}^{n}\rho_{iS_{2}}b_{iS_{2}}t_{iS_{2}}\right)\int_{L_{1}}^{L_{12}}{\left(y_{S_{2}}(x)\right)}^{2}dx\\ & +\frac{1}{2}\left(\sum_{i=1}^{p}\rho_{iS_{3}}b_{iS_{3}}t_{iS_{3}}\right)\int_{L_{12}}^{L_{123}}{\left(y_{S_{3}}(x)\right)}^{2}dx \end{array}$$

where *L*_12_ = *L*_1_ + *L*_2_, *L*_123_ = *L*_1_ + *L*_2_ + *L*_3_, and *θ* = 2π*f*. 

Based on Rayleigh method, we estimated the first bending resonant frequency of the equivalent resonator as:

(13) $$f_{r}=\frac{1}{2\pi }\sqrt{\frac{P_{m}}{K_{m}/\theta^{2}}}$$

The Euler-Bernoulli beam theory and Macaulay method are used to determine the deflections of the three sections of the equivalent resonator. The Macaulay method [23] is useful to describe different load types on structures that contain variable cross-sections [24]. Thus, the deflections along the three sections of the equivalent resonator can be obtained by:

(14) $${\left(EI_{z}\right)}_{S_{1}}\frac{\partial^{2}y_{S_{1}}(x)}{\partial x^{2}}=M_{S_{1}}(x)\;\;\;\;\;\;0<x<L_{1}\;{\left(EI_{z}\right)}_{S_{2}}\frac{\partial^{2}y_{S_{2}}(x)}{\partial x^{2}}=M_{S_{2}}(x)\;\;\;\;\;\;\;\;L_{1}<x<L_{12}\;{\left(EI_{z}\right)}_{S_{3}}\frac{\partial^{2}y_{S_{3}}(x)}{\partial x^{2}}=M_{S_{3}}(x)\;\;\;\;\;\;\;\;\;\;L_{12}<x<L_{123}$$

where *M_Sj_* is the bending moment of the *j*th section of the equivalent resonator and it can be calculated by integrating twice the load function of the resonator, which is determined by the Macaulay method.

The total load function of the equivalent resonator is given by:

(15) $$\begin{array}{ll} F(x)= & -M_{o}{\langle x-0\rangle }^{-2}+R_{o}{\langle x-0\rangle }^{-1}-\omega_{S_{1}}{\langle x-0\rangle }^{0}+\omega_{S_{1}}{\langle x-L_{1}\rangle }^{0}-\omega_{S_{2}}{\langle x-L_{1}\rangle }^{0}+\omega_{S_{2}}{\langle x-L_{12}\rangle }^{0}\\ & -\omega_{S_{3}}{\langle x-L_{12}\rangle }^{0}+\omega_{S_{3}}{\langle x-L_{123}\rangle }^{0} \end{array}$$

where *R_o_*, *M_o_* and $\omega_{S_{j}}$ are determined a:

(16) $$M_{o}=\sum_{i=1}^{3}\omega_{S_{j}}L_{S_{j}}$$

(17) $$R_{o}=\sum_{i-1}^{3}\omega_{S_{j}}L_{S_{j}}$$

(18) $$\omega_{S_{j}}=\sum_{i=1}^{3}\rho_{iS_{j}}gb_{iS_{j}}t_{iS_{j}}$$

where *g* is the gravitational acceleration and *ω_Sj_* is the weight per unit length of the *j*th section.

The deflection equation *y_Sj_*(x) of the equivalent resonator must satisfy the following boundary conditions:

(19) $$y_{S1}(0)=0\;\;\;\;\;\;\frac{\partial y_{S_{1}}(0)}{\partial x}=0\;y_{S_{1}}(L_{1})=y_{S_{2}}(L_{1})\;\;\;\;\;\;\;\;\;\;\frac{\partial y_{S_{1}}(L_{1})}{\partial x}=\frac{\partial y_{S_{2}}(L_{1})}{\partial x}\;y_{S_{2}}(L_{12})=y_{S_{3}}(L_{12})\;\;\;\;\;\;\;\;\;\;\;\frac{\partial y_{S_{2}}(L_{12})}{\partial x}=\frac{\partial y_{S_{3}}(L_{12})}{\partial x}$$

Based on Macaulay integration rules and integrating Equation (15) with respect to *x*, the shear load function *V(x)* of the equivalent resonator is given by:

(20) $$\begin{array}{ll} V(x)= & -M_{o}{\langle x-0\rangle }^{-1}+R_{o}{\langle x-0\rangle }^{0}-\omega_{S_{1}}{\langle x-0\rangle }^{1}+\omega_{S_{1}}{\langle x-L_{1}\rangle }^{1}-\omega_{S_{2}}{\langle x-L_{1}\rangle }^{1}+\omega_{S_{2}}{\langle x-L_{12}\rangle }^{1}\\ & -\omega_{S_{3}}{\langle x-L_{12}\rangle }^{1}+\omega_{S_{3}}{\langle x-L_{123}\rangle }^{1}+C_{1} \end{array}$$

By integrating the Equation (20) with respect to *x*, the bending moment function *M(x)* of the equivalent resonator is specified as:

(21) $$\begin{array}{ll} M(x)= & -M_{o}{\langle x-0\rangle }^{0}+R_{o}{\langle x-0\rangle }^{1}-\frac{1}{2}\omega_{S_{1}}{\langle x-0\rangle }^{2}+\frac{1}{2}\omega_{S_{1}}{\langle x-L_{1}\rangle }^{2}-\frac{1}{2}\omega_{S_{2}}{\langle x-L_{1}\rangle }^{2}\\ & +\frac{1}{2}\omega_{S_{2}}{\langle x-L_{12}\rangle }^{2}-\frac{1}{2}\omega_{S_{3}}{\langle x-L_{12}\rangle }^{2}+\frac{1}{2}\omega_{S_{3}}{\langle x-L_{123}\rangle }^{2}+C_{1}x+C_{2} \end{array}$$

To determine the magnitudes of the integration constants (*C*_1_ = *C*_2_ = 0), the boundary conditions (*V*(0) = *R*_o_ y *M*(0) = *M*_o_) at the clamped edge of the equivalent resonator are substituted into Equations (20) and (21). Next, the bending moment function in the three sections of the equivalent resonator is obtained through Equation (21):

(22) $$\begin{array}{ll} \mathrm{For}\;0<x<L_{1} & \\ & M_{S_{1}}(x)=-M_{o}{\langle x-0\rangle }^{0}+R_{o}{\langle x-0\rangle }^{1}-\frac{1}{2}\omega_{S_{1}}{\langle x-0\rangle }^{2}\\ \mathrm{For}\;L_{1}<x<L_{12} & \\ & M_{S_{2}}(x)=-M_{o}{\langle x-0\rangle }^{0}+R_{o}{\langle x-0\rangle }^{1}-\frac{1}{2}\omega_{S_{1}}{\langle x-0\rangle }^{2}+\frac{1}{2}\omega_{S_{1}}{\langle x-L_{1}\rangle }^{2}-\frac{1}{2}\omega_{S_{2}}{\langle x-L_{1}\rangle }^{2}\\ \mathrm{For}\;L_{12}<x<L_{123} & \\ & \begin{array}{ll} M_{S_{3}}(x)= & -M_{o}{\langle x-0\rangle }^{0}+R_{o}{\langle x-0\rangle }^{1}-\frac{1}{2}\omega_{S_{1}}{\langle x-0\rangle }^{2}+\frac{1}{2}\omega_{S_{1}}{\langle x-L_{1}\rangle }^{2}-\frac{1}{2}\omega_{S_{2}}{\langle x-L_{1}\rangle }^{2}\\ & +\frac{1}{2}\omega_{S_{2}}{\langle x-L_{12}\rangle }^{2}-\frac{1}{2}\omega_{S_{3}}{\langle x-L_{12}\rangle }^{2} \end{array} \end{array}$$

To obtain the static deflections *y_Sj_*(*x*) in the three sections of the equivalent resonator, Equation (22) are substituting into Equation (14) considering the boundary conditions expressed in Equation (19):

(23) $$\begin{array}{ll} \mathrm{For}\;0<x<L_{1} & \\ & y_{S_{1}}(x)=\frac{1}{{\left(EI_{z}\right)}_{S_{1}}}\left[-\frac{1}{2}M_{o}{\langle x-0\rangle }^{2}+\frac{1}{6}R_{o}{\langle x-0\rangle }^{3}-\frac{1}{24}\omega_{S_{1}}{\langle x-0\rangle }^{4}\right]\\ \mathrm{For}\;L_{1}<x<L_{12} & \\ & \begin{array}{ll} y_{S_{2}}(x)= & \frac{1}{{\left(EI_{z}\right)}_{S_{2}}}[-\frac{1}{2}M_{o}{\langle x-0\rangle }^{2}+\frac{1}{6}R_{o}{\langle x-0\rangle }^{3}-\frac{1}{24}\omega_{S_{1}}{\langle x-0\rangle }^{4}+\frac{1}{24}\omega_{S_{1}}{\langle x-L_{1}\rangle }^{4}\\ & -\frac{1}{24}\omega_{S_{2}}{\langle x-L_{1}\rangle }^{4}-\frac{1}{2}M_{o}L_{1}^{2}+\frac{1}{3}R_{o}L_{1}^{3}-\frac{1}{8}\omega_{S_{1}}L_{1}^{4}+\left(M_{o}L_{1}-\frac{1}{2}R_{o}L_{1}^{2}+\frac{1}{6}\omega_{S_{1}}L_{1}^{3}\right)x]\\ & +\frac{1}{{\left(EI_{z}\right)}_{S_{1}}}[\left(-M_{o}L_{1}+\frac{1}{2}R_{o}L_{1}^{2}-\frac{1}{6}\omega_{S_{1}}L_{1}^{3}\right)x+\frac{1}{2}M_{o}L_{1}^{2}-\frac{1}{3}R_{o}L_{1}^{3}+\frac{1}{8}\omega_{S_{1}}L_{1}^{4}] \end{array}\\ \mathrm{For}\;L_{12}<x<L_{123} & \\ & \begin{array}{l} y_{S_{3}}(x)=\frac{1}{{\left(EI_{z}\right)}_{S_{2}}}[-\frac{1}{2}M_{o}{\langle x-0\rangle }^{2}+\frac{1}{6}R_{o}{\langle x-0\rangle }^{3}-\frac{1}{24}\omega_{S_{1}}{\langle x-0\rangle }^{4}+\frac{1}{24}\omega_{S_{1}}{\langle x-L_{1}\rangle }^{4}\\ -\frac{1}{24}\omega_{S_{2}}{\langle x-L_{1}\rangle }^{4}+\frac{1}{24}\omega_{S_{2}}{\langle x-L_{12}\rangle }^{4}-\frac{1}{24}\omega_{S_{3}}{\langle x-L_{12}\rangle }^{4}+C_{3}x+C_{4}] \end{array} \end{array}$$

with

$$\begin{array}{ll} C_{3}= & \frac{{\left(EI_{z}\right)}_{S_{3}}}{{\left(EI_{z}\right)}_{S_{2}}}\left[-M_{o}L_{2}+\frac{1}{2}R_{o}L_{2}\left(2L_{1}+L_{2}\right)-\frac{1}{2}\omega_{S_{1}}L_{1}L_{2}L_{12}-\frac{1}{6}\omega_{S_{2}}L_{2}^{3}\right]+\frac{{\left(EI_{z}\right)}_{S_{3}}}{{\left(EI_{z}\right)}_{S_{1}}}\left[-M_{o}L_{1}+\frac{1}{2}R_{o}L_{1}^{2}-\frac{1}{6}\omega_{S_{1}}L_{1}^{3}\right]\\ & +M_{o}L_{12}-\frac{1}{2}R_{o}L_{12}^{2}+\frac{1}{6}\omega_{S1}L_{12}^{3}-\frac{1}{6}\omega_{S1}L_{2}^{3}+\frac{1}{6}\omega_{S2}L_{2}^{3} \end{array}$$

$$\begin{array}{ll} C_{4}= & \frac{{\left(EI_{z}\right)}_{S_{3}}}{{\left(EI_{z}\right)}_{S_{2}}}[\frac{1}{2}M_{o}L_{2}\left(2L_{1}+L_{2}\right)-\frac{1}{3}R_{o}L_{2}\left[3L_{1}L_{12}+L_{2}^{2}\right]+\frac{1}{12}\omega_{S_{1}}L_{1}L_{2}\left[3L_{1}\left(2L_{1}+3L_{2}\right)+4L_{2}^{2}\right]\\ & +\frac{1}{24}\omega_{S_{2}}L_{2}^{3}\left(4L_{1}+3L_{2}\right)]+\frac{{\left(EI_{z}\right)}_{S_{3}}}{{\left(EI_{z}\right)}_{S_{1}}}\left[\frac{1}{2}M_{o}L_{1}^{2}-\frac{1}{3}R_{o}L_{1}^{3}+\frac{1}{8}\omega_{S_{1}}L_{1}^{4}\right]-\frac{1}{2}M_{o}L_{12}^{2}+\frac{1}{3}R_{o}L_{12}^{3}\\ & -\frac{1}{24}\omega_{S_{1}}L_{1}\left[3L_{1}(L_{1}^{2}+4L_{1}L_{2}+6L_{2}^{2})+8L_{2}^{3}\right]-\frac{1}{24}\omega_{S_{2}}L_{2}^{3}(4L_{1}+3L_{2}) \end{array}$$

Finally, the first bending resonant frequency of the equivalent resonator is determined substituting the Equations (23) into Equations (11)–(13).

By considering small deflections of the pVEH microdevice at resonance, we estimate the dynamic deflections (*y_d_*) of the microdevice multiplying its static deflections (*y_sj_*) by the quality factor due to the air damping of the resonator [25]:

(24) $$y_{d}≅y_{sj}Q_{a}$$

Table 1 depicts the geometric parameters of the equivalent resonator layers used in the proposed mathematical model. In addition, Table 2 shows the values of the effective stiffness, weight per unit length, reaction load (*R_o_*) and bending moment (*M_o_*) of the equivalent resonator. Based on the values of the Table 1 and Table 2, the first bending resonant frequency of the equivalent resonator is 63.3 Hz.

### 2.3. Finite Element Method

**Figure 7:**
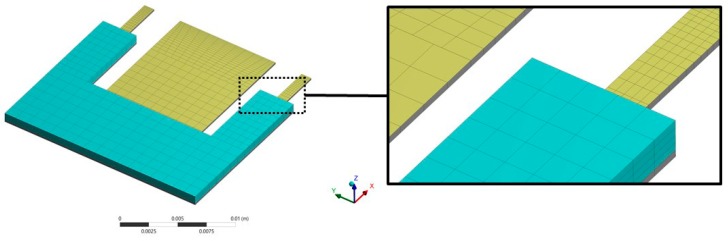
View of the mesh employed in the FEM model of the pVEH microdevice.

Finite element method (FEM) models of the pVEH microdevice are developed through ANSYS^®^ software to predict its electromechanical behavior. Figure 7 illustrates the mesh used in the FEM model of the pVEH microdevice, in which the properties of its materials, as shown in Table 3.

In this FEM model, the two aluminum electrodes are neglected due to that their thicknesses (100 nm) are smaller than the other layers of ZnO and PET. Table 4 indicates the piezoelectric matrix and piezoelectric dielectric matrix of the ZnO thin film, which are used in the FEM model.

**Figure 8:**
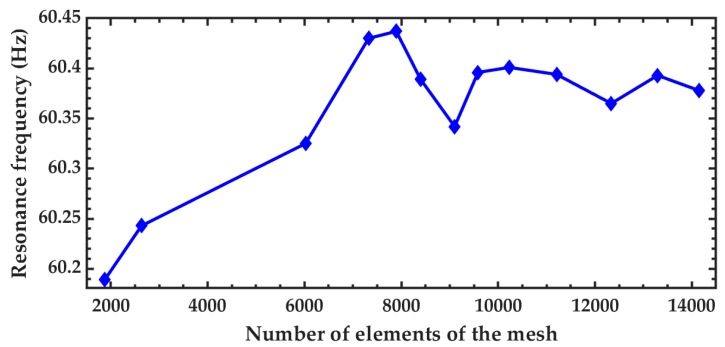
Variation of the resonant frequency of the pVEH microdevice as function of the number of elements used in the FEM model mesh.

**Figure 9:**
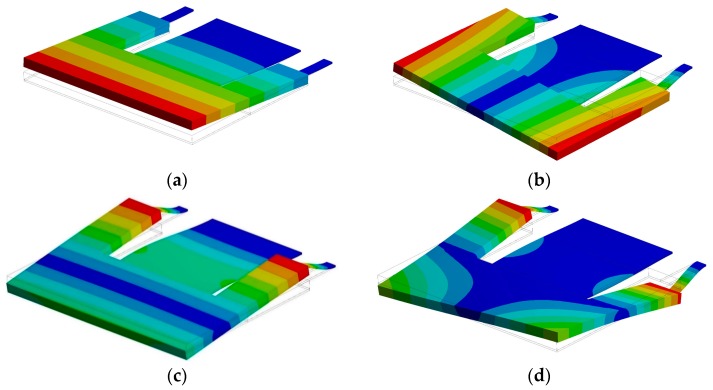
First four vibration modes of the pVEH microdevice: (**a**) first (60.3 Hz), (**b**) second (293.9 Hz), (**c**) third (751.3 Hz), and (**d**) fourth (2021.6 Hz) vibration mode.

Figure 8 depicts the variation of the resonance frequency of the first vibration mode of the microdevice using different number of elements in the mesh. This resonant frequency of the microdevice has small variations when the number of elements is higher than 9500. A modal analysis of the microdevice is performed using a FEM model to obtain its first four vibration modes (see Figure 9). The first vibration mode has a resonant frequency of 60.3 Hz and it is a bending mode (see Figure 9a). This resonant frequency has a relative difference of −4.9% with respect to that obtained by the analytical model. The second and third vibration mode (see Figure 9b,c) have frequencies of 278.9 and 728.4 Hz, respectively. Finally, the fourth vibration mode (see Figure 9d) has a resonant frequency of 1540.1 Hz and it registers irregular displacements.

**Figure 10:**
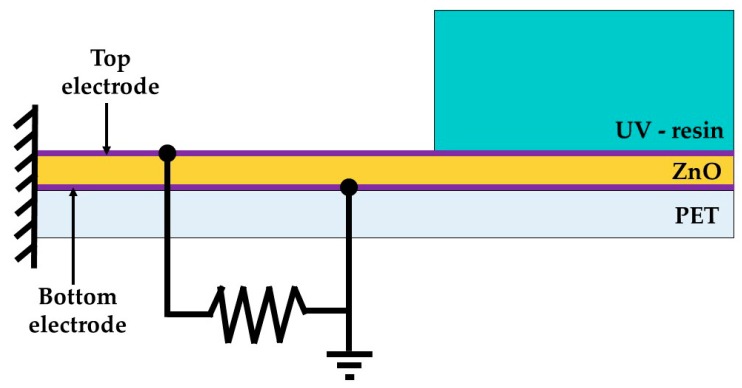
Schematic view of the electrical connection of the load resistance with the top and bottom ZnO surfaces used in the FEM model of the pVEH microdevice.

In the FEM model, a load resistance is considered in the electrical analysis of the pVEH microdevice. This resistance is developed using the element CIRCU94 with KEYOPT (1) = 0 through ANSYS software. The load resistance is connected to the top and bottoom ZnO surfaces, as shown in Figure 10. The optimal resistance value for the pVEH microdevice can be calculated with the following equation [26]:

(25) $$R_{opt}=\frac{1}{2\pi f_{r}C_{p}}$$

with

(26) $$C_{p}=\frac{\varepsilon_{0}\varepsilon_{33}bL}{h}$$

where *C_p_* is the capacitance of ZnO layer. 

## 3. Results and Discussion

**Figure 11:**
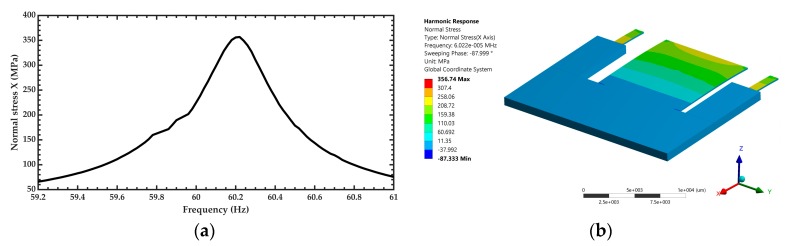
(**a**) Maximum normal stress along the *x* axis of the pVEH microdevice as function of its oscillation frequency and (**b**) distruibution of the normal stress along the *x* axis of the pVEH microdevice at resonance.

**Figure 12:**
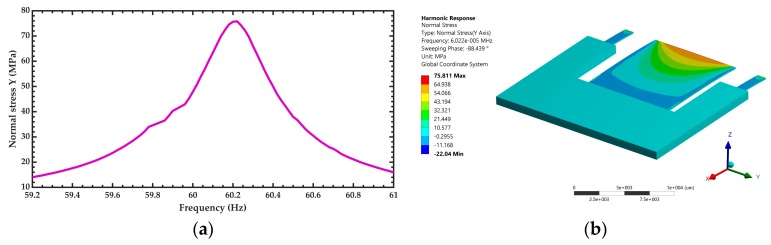
(**a**) Maximum normal stress along the *y* axis of the pVEH microdevice as function of its oscillation frequency and (**b**) distruibution of the normal stress along the *y* axis of the pVEH microdevice at resonance.

**Figure 13:**
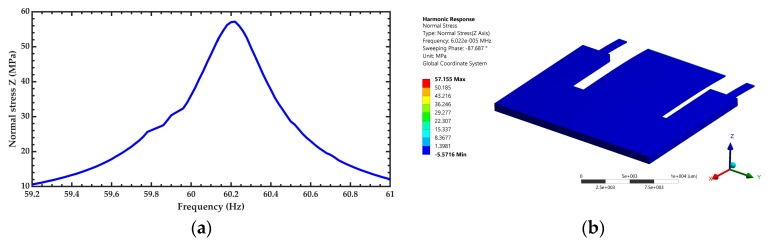
(**a**) Maximum normal stress along the *z* axis of the pVEH microdevice as function of its oscillation and (**b**) distruibution of the normal stress along the *z* axis of the pVEH microdevice at resonance.

**Figure 14:**
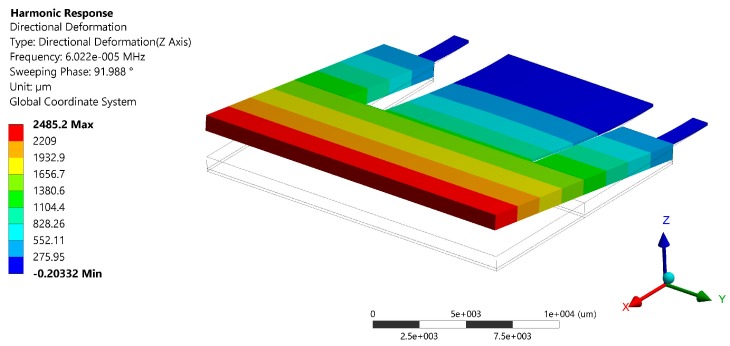
Displacements in the direction of the *z* axis of the pVEH microdevice at resonance.

**Figure 15:**
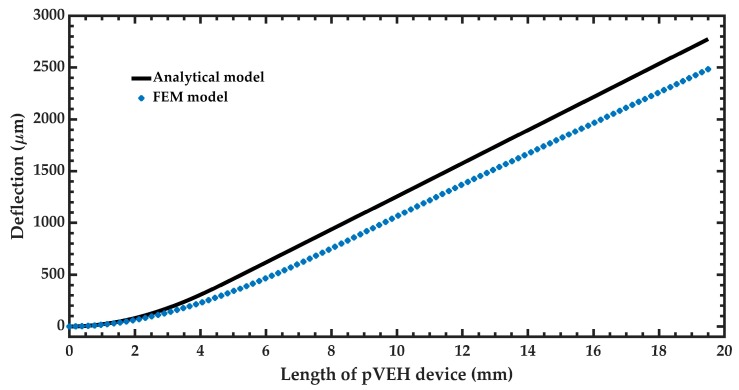
Maximum deflections (μm ) the pVEH microdevice as function of its length.

After of the modal analysis of the pVEH microdevice, an harmonic analisys of this microdevice is developed. For this analysis is used a frequency sweep from 59.2 to 61 Hz with increments of 0.018 Hz. This frequency range is around the first bending resonant frequency (60.3 Hz) of the pVEH microdevice. In the harmonic analysis is considered a quality factor of 213.58 related with the air damping of the pVEH microdevice. In addition, the FEM model of the pVEH microdevice is excited with a sinusoidal acceleration of 1.5 m/s^2^ along the *z* axis. This acceleration value had been reported in the mechanical vibrations of air conditioning vents at office buildings [14]. With this harmonic analysis is determined the mechanical stress, displacement and generated voltage of the microdevice. Figure 11, Figure 12 and Figure 13 show the normal stresses of the microdevice as function of its oscillation frequency. The maximum values of the normal stress are obtained at the first bending resonant frequency of the microdevice. The maximum magnitude of the nomal stress (356.74 MPa) along *x* axis is located on the top surface of the ZnO layer, which does not overcome its tensile strength of 412 MPa [27]. To reduce this maximum stress, a curved region can be designed on the connection area between the proof mass and ZnO layer. The maximum normal stress of the PET layer is 14.35 MPa, which is lower than its yield stress of 54.5 MPa [28]. On the other hand, the microdevice has a maximum displacement along *z* axis of 2485.2 μm (see Figure 14). This maximum displacement is registered along the free end of the microdevice when it oscillates at resonance. Figure 15 shows the maximum displacements of the microdevice at resonance using the analytical model and the FEM model. The response of the analytical model agree well with that of the FEM model.

**Figure 16:**
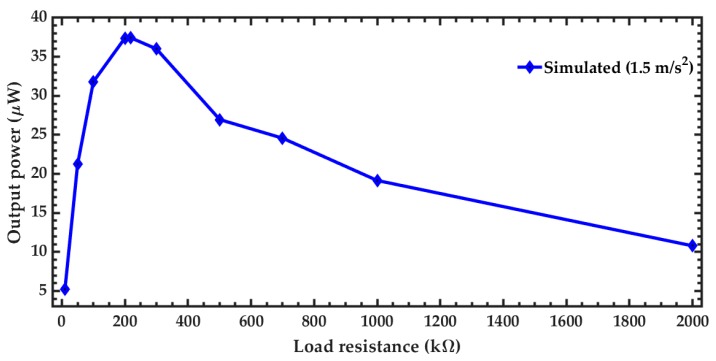
Output power generated by the pVEH microdevice as function of the oscillation frequency, considering a sinusoidal acceleration of 1.5 m/s^2^ along the *z* axis.

The variation of the output power of the pVEH microdevice is obtained using different load resistances from 10 to 2000 kΩ (Figure 16). The maximum output power of the microdevice is achieved with a load resistance around 210 kΩ. This value is close to the optimum load resistance (217.5 kΩ) obtained with Equation (25).

**Figure 17:**
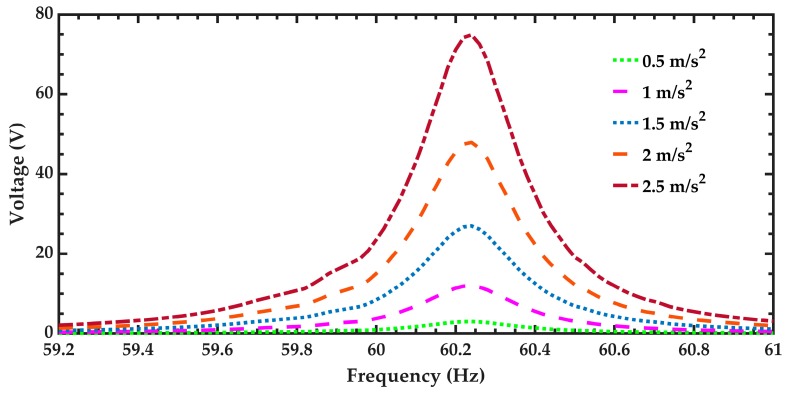
Output voltage generated by the pVEH microdevice as function of the oscillation frequency and considering five differents sinusoidal accelerations along the *z* axis.

**Figure 18:**
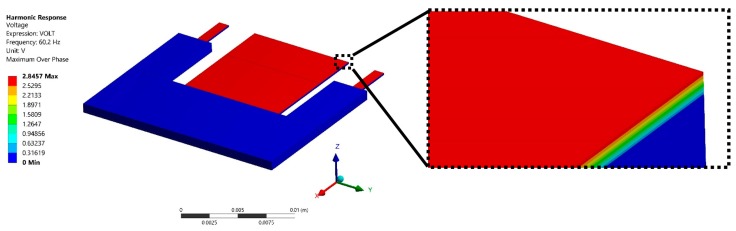
Dsitribution of the output voltage generated by the pVEH microdevice due to mechanical vibrations of ZnO layer and considering a sinusoidal acceleration of 1.5 m/s^2^ along the *z* axis.

The air flow inside the pipeline of the air conditioning vents could cause turbulence flow that alters the accelerations and vibration amplitudes of the air conditioning vents. In order to predict the electromechanical behavior of the pVEH microdevice under different aceleration amplitudes, five accelerations (0.5 m/s^2^, 1 m/s^2^, 1.5 m/s^2^, 2 m/s^2^, and 2.5 m/s^2^) along *z* axis are considered. For this case, the generated voltages by the pVEH microdevice are shown in Figure 17. The pVEH microdevice increments the generated voltages when the acceleration value increases. Future research works should include more studies about the effect of the air turbulence in the electromechanical performance of the pVEH microdevices. Figure 18 shows the voltage distribution of the pVEH microdevice when oscillates at resonance with an acceleration of 1.5 m/s^2^.

The output power by the pVEH microdevice can be defined as [29]:

(27) $$P=\frac{V_{rms}^{2}}{R_{opt}},$$

where *V_rms_* is the average voltage generated by the microdevice and *R_opt_* is the optimum resistance (217.5 kΩ) of the microdevice that is calculated with Equation (25).

**Figure 19:**
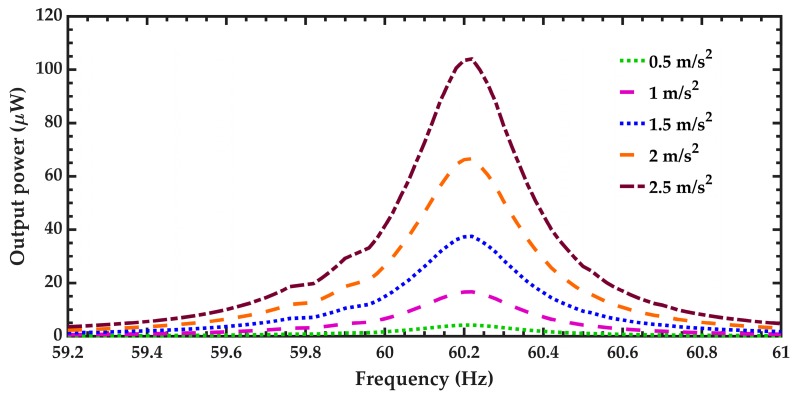
Output power generated by the pVEH microdevice as function of the oscillation frequency, considering five differents sinusoidal accelerations along the *z* axis and an optimal resistance of 217.5 kΩ.

Figure 19 illustrates the output power of the pVEH microdevice as function of the oscillation frequency and considering five different acceleration amplitudes. For an acceleration of 1.5 m/s^2^ (common acceleration value of air conditioning vents at office buildings), the pVEH microdevice at resonance can obtain an output power of 37.45 μW. Thus, the proposed pVEH microdevice can generate output voltage and power using the mechanical vibrations of air conditioning vents at office buildings. In addition, an array of pVEH microdevices connected in series can be employed to obtain more output voltage and power. This voltage could be used to supply electronic devices and sensors. 

## 4. Conclusions

The electromechanical modeling of a pVEH microdevice for air conditioning vents at office buildings is presented. This modeling considered the different multilayers and cross-sections of the microdevice resonator as well as the air damping. This microdevice is composed by a resonant structure of multilayer beams and U-shape proof mass of UV-resin (730 μm thickness). The multilayer beams has a flexible substrate (160 μm thickness) of polyethylene terephthalate (PET), two aluminum electrodes (100 nm thickness) and a ZnO layer (2 μm thickness). The first bending resonant frequency and deflections of the microdevice are determined using the Rayleigh and Macaulay methods, and the Euler-Bernoulli beam theory. Finite element method (FEM) models of the pVEH microdevice are developed to determine its first vibration modes, deflections, normal stresses and generated voltages. The results of the analytical model agreed well with those of the FEM models. The first bending resonant frequency of the microdevice obtained through FEM model has a relative difference of −4.9% with respect to that of the analytical model. The microdevice has a resonant frequency of 60.3 Hz, a maximum deflection of 2.485 mm considering an acceleration of 1.5 m/s^2^, an output voltage of 2.854 V, and generated power of 37.45 μW with a load resistance of 217.5 kΩ. An array of pVEH microdevices connected in series could be used to generate voltages using the mechanical vibrations of air conditioning vents at office buildings. These voltages could be supplied in electronic devices and sensors.

Future research works will include the fabrication and characterizaction of the pVEH microdevice. This characterization will consider the delamination study of the interface between PET and ZnO layers.

## Figures and Tables

**Table 1:** Geometric parameters of the equivalent resonator layers of the pVEH microdevice.

Geometric Parameter	Dimension (mm)	Geometric Parameter	Dimension (μm)
*L*_1_	4.5	*t*_1S1_ = *t*_1S2_ = *t*_1S3_	160
*L*_2_ = *L*_3_	7.5	*t*_2S1_ = *t*_2S2_ = *t*_2S3_	2
*b*_1S1_ = *b*_2S1_	12	*t*_3S1_ = *t*_3S2_	730
*b*_1S2_ = *b*_2S2_	17	*h*_1S1_ = *h*_1S2_ = *h*_1S3_	160
b_3S2_	7	*h*_2S1_ = *h*_2S2_ = *h*_2S3_	162
*b*_1S3_ = *b*_2S3_ = *b*_3S3_	20	*h*_3S1_ = *h*_3S2_	892

**Table 2:** Magnitudes of the effective stiffness, weight by unit length, reaction load and bending moment for the pVEH microdevice.

Parameter	Magnitude
(*EI_z_*)_S1_	22.421 × 10^−6^ N∙m^2^
(*EI_z_*)_S2_	1.6 × 10^−3^ N∙m^2^
(*EI_z_*)_S3_	13.2 × 10^−3^ N∙m^2^
*ω*_S1_	27.7 × 10^−3^ N∙m^−1^
*ω*_S2_	91.3 × 10^−3^ N∙m^−1^
*ω*_S3_	194.8 × 10^−3^ N∙m^−1^
*R*_o_	2.3 × 10^−3^ N
*M*_o_	28.94 × 10^−6^ N∙m

**Table 3:** Properties of the materials used in the FEM model of the pVEH microdevice [17].

Material Property	ZnO	PET Substrate	UV-Resin
Young’s module *E* (GPa)	137	2.4	2.4
Density *ρ* (kg/m^3^)	5665	1400	1037.78
Poisson ratio *ν*	0.25	0.36	0.34

**Table 4:** Material properties matrix of the piezoelectric ZnO thin film used in the FEM model of the pVEH microdevice [17].

ZnO piezoelectric stress matrix [*e*]
$[e]={\left[\begin{array}{ccc} 0 & 0 & -0.570878\\ 0 & 0 & -0.570878\\ 0 & 0 & 0.428446\\ 0 & 0 & 0\\ 0 & -0.480816 & 0\\ -0.480816 & 0 & 0 \end{array}\right]}_{6\times 3}{C/m}^{2}$
ZnO piezoelectric dielectric matrix [*ε_r_*] under the constant strain
$[\varepsilon_{r}]={\left[\begin{array}{ccc} 7.57 & 0 & 0\\ 0 & 7.57 & 0\\ 0 & 0 & 8.31 \end{array}\right]}_{3\times 3}$
